# Medical New York

**Published:** 1896-08

**Authors:** L. B. Grandy

**Affiliations:** New York Polyclinic


### CORRESPONDENCE.

### MEDICAL NEW YORK.

New York Polyclinic, July 10, 1896.

This is rather a dull season in medical New York. It is New York's vacation. The schools have closed, many of the professors are off at their summer homes or have gone abroad, the festive medical student has vanished, and even patients, I am told by medical friends, are distressingly scarce. But New York's great tenement-house multitude has no vacation; they are still here, and the hospitals and clinics and dispensaries continue to do a land-office business every day. The Polyclinic and Postgraduate schools are now in the midst of their summer sessions, the clinical lectures being delivered for the most part by the adjunct-professors; and while the physicians in attendance are few in nnmber at this season, it gives those who are present a better opportunity of examining and studying, under skilled direction, a great mass of interesting clinical material. With the Eye, Ear, Nose and Throat department of the Polyclinic, the Manhattan Eye and Ear Hospital, the New York Eye and Ear Infirmary, the New Amsterdam Eye and Ear Hospital, the Demilt Dispensary (Nose and Throat department), and other institutions of like character, the medical sojourner in New York, who is interested in the eye, ear, nose, and throat, need not want for work and study. In another letter I shall have something to say of the teachers and the teaching in these institutions.

### MORE LIGHT ON ANTITOXIN.

The New York Academy of Medicine has devoted two entire evenings of late to a discussion of the results obtained by the antitoxin treatment of diphtheria in hospital practice. Nothing was more noticeable in this discussion than the contrary, but conscientious, opinions which are still held by some of our best men. Dr. Joseph E. Winters, who has been perhaps the most vigorous opponent of the method in this country, presented an elaborate paper upon his own "Clinical Observations upon Antitoxin, and a Report of a Personal Investigation of this Treatment in the Principal Fever Hospitals of Europe during the Summer of 1895." He showed how the oft-quoted statistics which appeared to indicate such a remarkable reduction in the death-rate from diphtheria, were fallacious; how the disease varied in type and severity at different times; how a diagnosis of diphtheria was sometimes made when the disease was really not present, but recovery attributed nevertheless to the use of antitoxin; and how the use of antitoxin in some cases had not only been neither curative nor prophylactic, but had been injurious or even fatal in its results. He quoted from a number of well-known physicians in private practice who had tried and abandoned the treatment. He said also that those who have carefully watched this treatment in hospitals, and who formerly were earnest advocates of it, were now, after wider experience, opposed to it. He predicted that the time would come when every one would realize the truth of his position in regard to antitoxin.

At the same meeting of the Academy Dr. P. H. Ernst gave some of his "Personal Experience with Antitoxin." He reported seventy-seven cases of diphtheria, twelve treated with antitoxin and sixty-five without. He did not believe that antitoxin exerted any favorable influence whatever. Indeed, he thought that patients who recovered under this treatment had a more tardy convalescence than patients without it. A conservative paper was read by Dr. W. L. Stowell upon "Diphtheria with and without Antitoxin," in which it was shown that antitoxin could not claim better results than had been gotten by other methods of treatment. He quoted Jacobi, Huebner, Seibert, Rosenthal, Loeffler, and others, who had used formic chloride, carbolic acid injections into the tonsils, mercury, toluol, dioxide of hydrogen, and other well-known methods, with death-rates ranging from three to sixteen per cent. It was not claimed that antitoxin could do better than that.

On the other hand, Dr. W. H. Thomson read a paper, based upon late statistical compilations, giving the results obtained in a large number of hospitals. In 85 hospitals in differents parts of the world there had been 9,893 cases of diphtheria treated with antitoxin, and 1,820 deaths; and mortality of 18.3. In 53 of these hospitals, where the mortality had formerly been 44 per cent. 7,277 cases treated with antitoxin showed a death-rate of only 20 per cent. Dr. Thomson stated that the verdict of the medical world, after a trial of one year and a half, was favorable to antitoxin, and he believed that it marked a great advance in the practice of medicine.

In the discussion of these papers Dr. John W. Brannan vigorously attacked the statements and conclusions of Dr. Winters, particularly as regards the experience of antitoxin in the Willard Parker hospital. He showed the fallacies also in several of Dr. Winters' quotations from foreign hospitals. Dr. A. H. Smith presented some figures from boards of health in this country, giving report of 5,120 cases treated with antitoxin, and a mortality of 10 per cent.; formerly in the same cities the mortality had averaged 40 per cent. He was in favor of the treatment. Dr. Berg stated that while hospital statistics were not reliable, he was a believer in antitoxin from his experience in private practice. Dr. W. H. Park agreed with Dr. Brannan, and disagreed with Dr. Winters as to the results obtained at the Willard Parker hospital by the antitoxin.

A composite opinion of the members of the Academy who have taken part in the discussions upon antitoxin is certainly favorable to this method of treating diphtheria. Some members still prefer to take a conservative stand, awaiting further developments, and doubtless this is wisest at present. Indeed, in personal conversation with medical friends here, I find many of them who think the value of antitoxin is still *sub judice*.

Simultaneous with this discussion in the Academy comes the report of the American Pediatric Society's Collective Investigation into the Use of Antitoxin in Private Practice (made at the late meeting of the Society in Montreal, May 26). Circulars sent out by the Society in April met with responses from 613 physicians in 114 cities, in fifteen States, the District of Columbia, and Canada. Cases were also reported from the Health Departments of New York City and Chicago. There was a grand total of 5,794 cases, with 713 deaths, or a mortality of 12.3 per cent. Of these cases 218 were moribund at the time of injection, and died within twenty-four hours. If these be excluded, the mortality is reduced to 8.8 per cent. Of 4,120 cases injected during the first three days there were 303 deaths—a death-rate of 7.3 per cent. If from this number cases be deducted which were moribund at the time of injection, the mortality becomes 4.8 per cent. The report emphasizes the importance of early injections, within the first three days. When the larynx is involved in the diphtheritic process, the effect of the serum has been to arrest the extension of the membrane. This was noted by more than 350 physicians. Therefore it has been necessary to resort to intubation or tracheotomy less often than when antitoxin was not used. Moreover, in the experience of several operators when the use of antitoxin and intubation were both practiced on the same patients, the mortality has been only half as great as when intubation alone was done. It appears, then, that the serum has saved double the number of cases of laryngeal diphtheria that have been saved by any other method of treatment. In the majority of cases reported only one dose (injection) was given; in severe cases, two or three.

More than 600 of the physicians contributing to this report are strongly in favor of the treatment, the majority enthusiastic over it. There was no evidence to show that antitoxin was productive of any harmful results in the way of broncho-pneumonia, cardiac depression, nephritis, or disturbances of the nervous system.

The friends of the antitoxin treatment certainly have good reason for encouragement in this report. The committee of the Society will continue their work for another year, and they solicit the further coöperation of the profession in their endeavor to arrive at the truth. The Society's first report is published as a supplement to *Pediatrics* for July, to which the reader is referred for details.

### X-RAY PHOTOGRAPHY.

I had the pleasure, several evenings ago, of attending a very interesting X-ray *séance* at a meeting of the Brooklyn Medical Society. I was more than surprised at the demonstration. Professor William Stubenbord, of New York, first read a short paper upon the history of this discovery by Roentgen and of its subsequent study and development. He then exhibited a handsome and highly instructive series of X-ray photographs, or skiagraphs, which he had made on various parts of the body, normal and diseased, several of which attested the value of this procedure in surgical diagnosis. One skiagraph was exhibited, of an entire person, taken in four sections; the bony skeleton was complete and distinct, and showed unmistakably a lateral curvature of the spine. A female pelvis was also shown, and the possibilities of this method in detecting deformities of the pelvis became manifest. An old periostitis of the lower extremity of the femur, which had escaped other methods of detection, was also exhibited. A picture of a fractured metacarpal bone, taken through the splints and bandages, was presented. By this method, then, the surgeon can now tell whether or not the ends of fractured bones are in apposition, as they were in the above case. Next there was a beautiful demonstration of this "photography" upon a case present. A boy about twelve years of age had been shot some months ago, the pistol-ball entering through the outer extremity of the right eyebrow. All efforts to locate the ball proved fruitless, though it was supposed to be situated somewhere in the frontal sinus. Soon vision was lost in the corresponding eye. Here was a good case for the X-ray. The boy was laid on his right side on a table, with his head on a hard-rubber plate, which covered over the sensitive photographic plate. A strong static electrical machine was then set in motion, and copper wires were attached to the two poles. The other ends of these wires were then attached to the poles of a Crookes' vacuum tube, or bulb, and the latter made stationary about sixteen inches above the patient's head. The electric current produces in this bulb an appearance more like a phosphorescent glow than a bright light. This "light" is now called the "cathodal streamer." After an exposure of thirty minutes the photographic negative was developed by the usual process, and a round object was discovered in a position which was thought to be between the eyeball and outer wall of the orbit.

The entire demonstration of the evening was full of the greatest interest for the medical spectators, both from the scientific interest attaching to this marvelous discovery and from the avenues of possible usefulness which it opens up as a method of surgical diagnosis. The collection of skiagraphs, as well as the practical application of the process, as presented at this meeting, was said by the President of the Society to be the best X-ray exhibition yet made in this country.

It must be remembered that this discovery is as yet only seven months old. No doubt it will be rapidly studied and perfected, and however valuable it may be even now as a diagnostic resource for the surgeon, it will certainly become more and more so as our knowledge of it is extended.

Since the above was written Dr. Stubenbord has kindly shown me in his office the finished photograph of the boy's head, with the bullet clearly outlined in the position above named. An operation will soon be made to remove the bullet. Dr. Stubenbord has done more than any one else in this country to bring the Roentgen photography to the aid of the surgeon. His office is elegantly fitted up for this purpose. He is now preparing, by request, an elaborate paper on the subject for the Academy of Medicine. Cases of foreign bodies in the tissues, or of suspected bone or joint disease or deformity are submitted to him almost every day for diagnosis. He is enthusiastic over the new photography, and believes that it is as yet only at the beginning of its usefulness.

L. B. GRANDY, M.D.

